# The Sanitary Institute

**Published:** 1904-07-23

**Authors:** 


### THE SANITARY INSTITUTE.

COMMUNICATED.

The Sanitary Institute, which is holding its twenty-second Congress in Glasgow this year, was formed in 1876. At that time the public were ignorant and indifferent so far as sanitation was concerned. The bulk of the members of the medical profession were sceptical as to the value of sanitation, and disinclined to recognise it as a force, by the utilisation of which they could materially improve the public health. Very few houses were properly drained; most of the Government offices, and even the Queen's Palaces, were very insanitary. There was an absence of provision for the isolation of infectious diseases and the adequate treatment of infected patients. The water supply nearly everywhere was indifferent or defective, and the systems of drainage in many places were bad. The majority of the hospitals and asylums had no plans of their drains, and where plans existed they were far from accurate, and in no way to be relied upon. The mortality in the best hospitals after serious surgical operations was 37·8 per cent. There was little literature on the subject of sanitation, and that which existed was mostly buried in Blue Books not readily accessible. There was no test of competence, and no means of training persons as sanitary officials. Local surveyors, inspectors of nuisances, and even medical officers of health were all without special training or qualification, and did not possess accurate knowledge or information.

Such, briefly, is a description of the state of matters which existed 21 years ago, as drawn by Sir Henry Burdett, one of the founders of the Sanitary Institute.

One of the first steps taken by the Sanitary Institute was to establish examinations for local surveyors and inspectors of nuisances. The first examination was held in October 1877, when eight candidates came forward. These examinations gradually gained in public estimation, and sanitary authorities began to recognise the advantage they afforded in guiding them in the selection of sanitary officials. The examinations revealed the fact that candidates for appointment as sanitary officers had considerable difficulty in obtaining knowledge of the principles of sanitation, and the Council established systematic courses of instruction suitable for these officers. The examinations, which had hitherto been held in London only, were, in 1890, extended to the provinces, so as to make them more accessible to candidates living at a distance. In the following year the training lectures for sanitary officers were also extended to the provinces, and arranged in conjunction with the County Councils; and in the Public Health (London) Act, the Institute obtained the insertion of a clause requiring all sanitary inspectors in London, appointed after January 1st, 1895, to hold a certificate of qualification.

In 1892 arrangements were made for adding practical demonstrations to the courses of lectures for sanitary officers, and visits for the students were arranged to sewage works, waterworks, trade premises, and other places that would be instructive from a sanitary point of view. In the following year these practical demonstrations were considerably extended and more elaborately organised. A first course of lectures on the sanitation of industries and occupations was held, and various experimental investigations were carried out.

The lectures and meetings have been extended over England and into Scotland and Ireland.

The Parkes Museum of Hygiene was instituted in the same year as the Sanitary Institute, as a permanent memorial of Dr. Edmund Alexander Parkes, the first Professor of Hygiene at Netley. The Museum is largely used by the London Hospital Medical Schools and Colleges for their classes for the diploma in Public Health, and is recognised by the Science and Art Department in connection with their classes, and the class visits paid by students to the Museum may be counted as a class attendance for the purpose of the examination grant. Visits are frequently made to the Museum by representatives from the Colonies and the Continent, with the object of obtaining information for establishing or enlarging similar museums.

In order still further to aid in the diffusion of sanitary knowledge, meetings are held for the reading and discussion of papers; there is a library containing works on hygiene and sanitation, Parliamentary and other official Reports and Transactions of other Societies; and the Transactions of the Institute are published in the form of a quarterly journal. But the Congresses, which are arranged to take place in provincial towns, so as to bring together those interested in sanitary science to read papers and discuss questions affecting the public health, are looked upon as a most important means of spreading the advantages of the Institute throughout the country.

The number of members and associates of the Institute is now over 3,300. Four hundred authorities throughout the kingdom and a large number of societies appoint delegates to attend the Congress. The total attendance is usually about 2,000.

In 1896 his Royal Highness the late Duke of Cambridge became President of the Institute. The President elect is his Grace the Duke of Northumberland, K.G.

The President of the Glasgow Congress will be the Right Hon. Lord Blythswood.

